# Form follows function: pragmatic controlled trials (PCTs) have to answer different questions and require different designs than randomized controlled trials (RCTs)

**DOI:** 10.1007/s10389-012-0544-5

**Published:** 2012-11-07

**Authors:** Franz Porzsolt, Martin Eisemann, Michael Habs, Peter Wyer

**Affiliations:** 1 Clinical Economics at the Institute of History, Philosophy and Ethics in Medicine, University of Ulm, Frauensteige 6, 89075 Ulm, Germany; 2 Faculty of Health Sciences, Department of Psychology, University of Tromsø, 9037 Tromsø, Norway; 3 Associate Professor of Pharmacology Medical Faculty, Ludwig Maximilian University Munich, 80539 Munich, Germany; 4 Associate Clinical Professor of Medicine, Columbia University College of Physicians and Surgeons, New York, NY 10032 USA

**Keywords:** Randomized controlled trial (RCT), Pragmatic controlled trial (PCT), Public health research, Comparative effectiveness research, Internal validity, External validity

## Abstract

**Aim:**

Rising concern for demonstrated real world comparative effectiveness has heightened interest in “pragmatic trials” design. Pragmatic trials investigate whether the efficacy, presumed or found in explanatory trials under ideal conditions, can also be detected under real world conditions, i.e. effectiveness. It is also recognized that ‘real world’ effects which are usually addressed in public health research gain growing interest in confirming the ‘road capability’ of results obtained under ideal study conditions. This paper demonstrates that studies under ideal or real world conditions use different methods, generate different information and cannot replace each other.

**Subjects and methods:**

The PCT design meets four requirements of public health and of effectiveness research. It includes all individuals who presented with the selected condition. It classifies the included individuals according to baseline risks. It enables plausibility controls. Finally, it compares the outcomes resulting from specified and not-specified interventions or treatments.

**Proposal:**

We propose a pragmatic controlled trial (PCT) design in which patient preference and other co-factors crucial in determining the actual effectiveness of interventional options will not be neutralized by concealed randomization and blinding. This design is applicable to record the selected interventions and generated outcomes in day-to-day health care and is capable of incorporating preference and other participative factors into assessment of effectiveness.

**Conclusions:**

The PCT design is useful for public health research, e.g. the effectiveness of interventions to change smoking habits or to prevent death from breast cancer, as well as for comparative effectiveness research where it will supplement the traditional randomized controlled trial (RCT).

## Background

Randomized controlled trials (RCTs) are frequently considered the gold standard of clinical research and superior to non-randomized trials. Accordingly, strictness of randomization, concealment of allocation and extent of blinding are considered to constitute the principles of the quality of clinical trials (Jadad et al. 1996). This position may be problematic if a RCT is not the optimal method to answer a certain research question. In this case, researchers are prevented from deliberately and carefully selecting the specific optimal study design and therefore violate an important principle of scientific research, i.e. the coherence of trial design and study purpose, which is well established within the methodological literature (Spilker 1991).

Discussions of the relative merits of ‘explanatory’ and ‘pragmatic’ trials, a distinction introduced by Schwartz and Lellouch (1967), frequently revolve around issues of similarity of research to real world conditions with respect to patients, interventions, comparisons and outcomes (Karanicolas et al. 2009; Oxman et al. 2007) However, Schwartz and Lellouch (1967) emphasized that the two types of trial correspond to two distinct objectives, with ‘explanatory’—ideally a RCT—trials seeking answers to the question of whether an intervention “can work”, in contrast to ‘pragmatic’ trials seeking answers to the question of whether the intervention “does work” in the context of actual clinical practice. In a recent published exchange of ideas, one commentator suggested that Schwartz and Lellouch’s concept be abandoned in favour of a distinction between trials that investigate the biological mechanism and trials that aim for practical application (Karanicolas et al. 2009). This proposal was criticized as obscuring Schwartz and Lellouch’s differentiation between the objectives of different categories of a clinical trial, all of which focus on clinical outcomes related to interventions (Oxman et al. 2009).

The aforementioned proposals and controversies have been confined to contrasting visions of research objectives and the practical application of randomized trials. They have not considered aspects of a pragmatic trial design and objectives that are incompatible with randomization and blinding. As pointed out by Oxman et al. (2009), the issues in these debates are largely reducible to matters of applicability of randomized trials to practice and policy decisions. For example, the nature and extent of patient selection, the intensity of monitoring and the level of compliance of both patients and practitioners with the study protocol, including compliance with medications, may be a matter of degree. Indeed, the discussions are pervaded by the notion that the distinction between explanatory and pragmatic defines a continuous spectrum (Thorpe et al. 2009; Sackett 2006). Thorpe et al. (2009) propose a rating tool for the purpose of locating a particular trial or design on such a spectrum.

Clinical researchers have recognized that RCTs are not sufficient to answer all important questions (Schwartz and Lellouch 1967; Karanicolas et al. 2009; Oxman et al. 2007). Public health researchers have recently emphasized that adequate alternatives to RCTs are needed in public health research (Bonell et al. 2011; Cousens et al. 2011). Clinical research is usually testing a hypothesis, e.g. comparing two treatments by designing an ideal experiment, e.g. a concealed and blinded randomized trial. By using this approach, effects can be excluded which are considered as confounders such as doctor and patient expectations, preferences and placebo effects. Public health research usually analyzes observed coincidences and investigates a possible causal relationship. This research does not exclude doctor and patient expectations, preferences and placebo effects. New approaches therefore propose advanced statistical methods to identify and explain the variance in non-randomized trials (Cousens et al. 2011; Porzsolt et al. 2012). Explanatory trials can demonstrate efficacy, i.e. whether a principle would work under ideal conditions, but it cannot demonstrate effectiveness, i.e. whether the same principle would also work under real world conditions. This second effect can only be detected in pragmatic trials.

Two examples demonstrate that this conflict of results obtained under ideal versus real world conditions is a current topic in medical research—the efficacy of nicotine replacement therapy had been confirmed some years ago in a Cochrane review-based on randomized trials (Stead et al. 2008), while the opposite effect was found in a cohort study describing the effectiveness of such interventions (Alpert et al. 2012). A comparable conflict is ongoing in breast cancer screening. Recent articles usually based on cleverly designed pragmatic trials (Zahl et al. 2008; Zahl et al. 2011) express considerable doubts on the value of breast cancer screening. The details behind explanatory and pragmatic trial designs are addressed in the present paper.

## Pragmatic controlled trials (PCTs) consider expectations, preferences and ‘placebo effects’

The aforementioned discussions are pertinent to the issue of the applicability of conclusions drawn from randomized trials to healthcare decisions made under practical conditions. However, they do not address the broader issue of when randomization and blinding do, and do not, reflect a study design appropriate to the objectives of pragmatic trials. Specifically, the roles of patient expectation, patient preference and the so-called placebo effect may in many situations play a decisive role in determining the effectiveness of an intervention aimed at improving patient outcomes in actual care settings. Treatment preferences of doctors, nurses and patients are different and are likely to have different impact on the final treatment decisions. Such preferences can in fact be measured by methods such as conjoint analysis, but require complex and intensive studies (Porzsolt et al. 2010a). Such studies are common in market research but are rarely completed in health care research. Even if the reported preferences in mind can be identified in preference studies, one cannot exclude that the communicated theoretical preferences will be identical to the options that have to be selected under real conditions. More important than the identification of the hypothetical preferences of patients, doctors and nurses might be the option that will finally be selected. Hence, research with the objective of illuminating the practical effectiveness of such interventions must choose a design that incorporates these ‘co-factors’. In such situations, randomization and blinding must yield to an alternative design.

Waber et al. (2008) elegantly demonstrated the potential role of patient expectation in determining outcomes by using 82 medical students as subjects. The students were informed that they would receive different pain-killers to control pain in their feet induced by electrical strikes. The probands, in fact, received the same placebo but reported different degrees of pain relief depending upon the type of information provided. This experiment demonstrated that expectations induced by information might influence the comparative effectiveness of a pain treatment. However, this experiment cannot be replicated among patients for ethical reasons. More broadly, Brewin and Bradley (1989) compellingly described the circumstances under which patient preferences are inherently bound to potential effectiveness. In particular, in the case of counselling interventions, and other interventions requiring active participation of the patient, it is well established that patient preference and willingness constitute an inherently necessary condition for effectiveness (Brewin and Bradley 1989). Hence, a design randomizing patients to a treatment mode, irrespective of their preferences, could not, by definition, yield results relevant to real world effectiveness. Finally, Kaptchuk et al. (2008), in a three-arm randomized trial, demonstrated a ‘dose-response’ effect on quality of life outcomes of what otherwise might be called the ‘placebo’ effect. While their design did not directly address patient or practitioner preferences, their observations suggest that anticipation, expectation and clinical interaction constitute strong determinants of real world effectiveness, i.e., the bonding of preference between practitioner and patient for a treatment may well influence the magnitude of the ‘placebo’ effect achieved in association with a treatment choice.

**Fig. 1:**
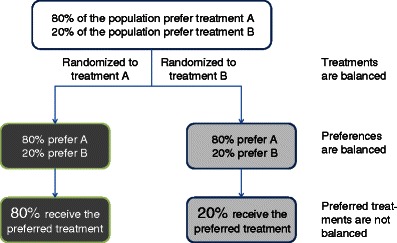
The randomization will generate different groups of patients unless the patients’ preferences are equally distributed, i.e. 50:50, in the randomized population. If distributed unequally, as described in the example, rather huge differences may occur in the proportions of patients who receive the preferred treatment option

Practitioners and patients with strong preferences may refuse participation in a randomized trial and consequently will increase the risk of sampling bias. Patients with weak preferences will increase the risk of performance bias as those who receive their preferred treatment will more benefit from treatment than those who will get the not-preferred treatment. Figure 1 illustrates that these ‘preference-based effects’ can only be avoided if either the same proportions of patients prefer one of the choices or the treatments can successfully be blinded. It is interesting that systematic reviews which explicitly searched for ‘preference-based effects’ could not confirm the postulated results (Stengel et al. 2006; King et al. 2005). This is likely attributable to the fact that most preference-based trials have conformed to a design in which allocation by preference follows initial acceptance or rejection of randomization (Stengel et al 2006; Porzsolt and Stengel 2006). Hence, patients with a strong preference for one of the therapeutic options were likely to reject randomization, thereby masking the preference effect across the study arms. These experiments confirm that it is rather difficult to quantify ‘preference-based effects’.

In summary, the conventional design of trials utilizing randomization, concealment of allocation and blinding to treatment arm with the objective to test efficacy of interventions is likely to fail to accurately assess real world effectiveness due its inability to capture the multiple mechanisms contributing to clinical outcomes. Although such trials may incorporate some features of a pragmatic or ‘real world’ orientation, such as broad patient selection, laxity on compliance controls and intensity of monitoring, preference-based effects will be distorted or obscured. Although shared preference for therapeutic options will generally increase the real world effectiveness, other practical issues such as co-morbidity, patient and practitioner compliance may decrease it. Accordingly, a trial design is required which is capable of capturing most real-world issues, including preference-based effects, in order to fully assess effectiveness. We here describe the outline of three-arm non-randomized pragmatic controlled-trial design.

## Proposal for a pragmatic controlled trial design

**Fig. 2:**
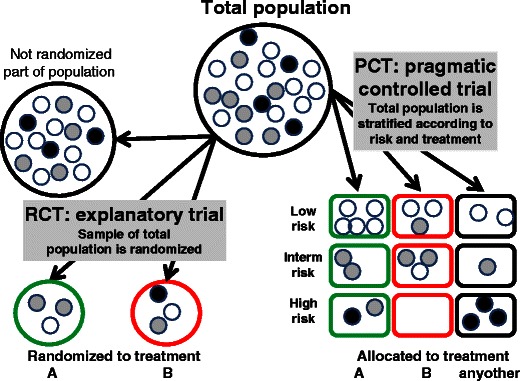
Differences of randomized controlled trials (*RCT*) and pragmatic controlled trials (*PCT*). It is shown that a particular total population of patients (e.g. in a hospital or clinic) will include patients with high risk (*black*), intermediate (*gray*) and low risks (*white*) for a condition that should be prevented by the selected treatments (e.g. hospitalization of more than two weeks or death). A detailed description of the differences between RCTs and PCTs is given in the text

The proposed study design, appropriate for demonstration of the effectiveness, is shown in Fig.2 and is named pragmatic controlled trial (PCT). In a PCT, data of all patients of the total service population are included in the evaluation. The suitability of this step has eventually been approved by an institutional review board. By inclusion of all patients, the risk of selection bias is reduced. The administered treatment will not randomly be selected but will be based on existing preferences of doctors and patients. To maintain some control over the effect of different baseline risks on outcome, the patients are allocated to high, intermediate, and low risk groups respectively, using the best prognostic criteria available such as validated prediction rules. The selection of prognostic criteria will depend on the investigated outcome, i.e., the assessment of different outcomes requires consideration of different prognostic criteria. Different treatments are then compared within identical risk strata (horizontal comparison in Fig. 2). An important advantage of the design is that the accuracy of the selected risk criteria can be verified by comparing the vertical groups in Fig. 2. Low risk patients and high risk patients who receive the same treatments should demonstrate different outcomes.

The first two of the three study arms of the PCT investigate the clinical outcomes of two favored treatment options applied under day-to-day conditions. This means that the selection of the treatment should be based on the information ideally derived from explanatory trials and on the doctors and patients preferences. The third study arm in a pragmatic trial has to guarantee that the outcomes of all treated patients are reported in order to reduce the risk of selection bias. Therefore, the third study arm should include all eligible patients who were seen in the participating institution during the study period except those patients who are reported in one of the first two study arms. This inclusion criterion represents an important difference between explanatory and pragmatic trials.

If the pragmatic controlled trial fails to confirm a difference between the two investigated treatments (A and B in Fig. 2), the results of the two investigated treatments should be compared with the third study arm. This additional step is necessary to test the specificity of the result. It has to be demonstrated that these two treatments—despite of similar effectiveness—generate better outcomes than the average of any other treatments (in the mixed third group).

The similarities and differences between the three-arm PCT design we are proposing and the conventional randomized explanatory trial are highlighted in Table 1. Six of the ten steps (step Nos. 1, 2, 3, 7, 8 and 10) are identical in explanatory and pragmatic trials. The remaining four steps (step Nos. 4, 5, 6 and 9) are similar. However, it is worth mentioning that the sequence of step Nos. 4–6 differs between explanatory and pragmatic trials. These ten steps guide to implement the appropriate study design (Porzsolt et al. 2010b).

**Table 1:** The recommended steps in explanatory and pragmatic trials

Step Nos.	Explanatory trial	Pragmatic trial
1	Define the type of research (explanatory or pragmatic trial) that should be completed	
2	Phrase the four parts of the precise study question according to the principles of Evidence-Based Medicine	
3	Select the most appropriate study design (among explanatory or pragmatic designs)	
4	Define inclusion/exclusion criteria and the investigated treatment options	Define patient risk groups and the investigated treatment options
5	Ask eligible patients to sign the informed consent for randomization, blinding, treatment, evaluation and publication of data	Allocate all patients according to doctors‘ and patients‘ preferences to treatment options or to “other treatment”
6	Randomize the eligible patients who signed the informed consent to the selected treatment options	Ask all patients to sign the informed consent for evaluation and publication of the data
7	Assure that the follow up period is long enough to observe a sufficiently large number of study endpoints	
8	Demonstrate in ‘Table 1’ the similar distribution of baseline risks within the compared study groups	
9	Analyze the results according to the ITT principle	Analyze the results according to Bayesian Statistics
10	Use appropriate statistics to confirm non-random effects.	

*ITT* intention-to-treat principle. Neither the topics (colors) nor the sequence of the topics are identical in explanatory and pragmatic trials indicating that both, content and sequence of the steps are different

## Discussion

The research design described in this paper is based on the concept “Form Follows Function” that was developed as a guide for the construction of the first skyscrapers in Chicago (Sullivan 1896). This concept is applied to two types of research, explanatory and pragmatic trials to different research areas, clinical research and public health research and to different clinical trials which intend to measure two outcomes under two different conditions. According to the* Framework for Design and Evaluation of Complex Interventions to Improve Health* published by the UK Medical Research Council (Campbell et al. 2000), a clinical trial can only be effectively implemented if the problem to be solved has been properly addressed. Despite the persistent problem concerning an exact definition of the essential characteristics of a clinical trial (Wright et al. 2008), we can conclude that the quality of a study will definitely be compromised if the stated study question is not absolutely clear. The two basic categories of questions that are asked in clinical medicine are:

- “Does a new principle work at all under ideal study conditions, i.e. is it efficacious”?
- “Will the efficacious principle do under real world conditions what it promised to do under ideal study conditions, i.e. is it effective ”?

Consequently, explanatory trials measure efficacy under ideal but artificial conditions, while pragmatic trials measure effectiveness under real day-to-day conditions. For clarity reasons, we do not consider any other, e.g., mixed conditions. When extracting the essential steps of experimental and pragmatic trials, one will essentially end up with the steps described in Table 1 and Fig. 2, which illustrate several important differences.

The pragmatic trial design is particularly pertinent in the investigation of effectiveness of interventions affecting public health. Screening interventions and interventions to modify individual susceptibility to disease are subject to public debate and controversy and therefore likely to be associated with strong patient and practitioner preferences, which in turn may affect outcome. A notable example is that of breast cancer screening. Recent recommendations against routine screening for women under age 40 on the part of an independent guideline group in the United States were met by divisive and polarized debate and corresponding coverage in the popular press (Quanstrum and Hayward 2010).

The efficacy assessed in an explanatory trial must not be misinterpreted as a pure effect mediated by a specific treatment. Like effectiveness, efficacy is a mixed effect caused by a specific intervention and by confounders. The difference between efficacy and effectiveness is that the confounders in experimental and control groups are (ideally) identical in an explanatory trial but may be different in a pragmatic trial. By definition of the inclusion and exclusion criteria, the number of confounders is reduced in an explanatory trial in contrast to a pragmatic trial in which the access is not limited by these criteria. Testing the external validity of an effect—presuming that the internal validity is known—requires several pragmatic trials as a large number of conditional and environmental factors has to be controlled (Di Blasi et al. 2001). The randomization in explanatory trials is replaced by stratification according to risk groups in pragmatic trials. Finally, the intent-to-treat analysis in explanatory trials is replaced by Bayesian statistics in pragmatic trials. This means that patients should be evaluated in the treatment groups to which they were allocated at the start of treatment even if the treatment was subsequently changed.

If these simple instructions for the planning and conduct of clinical trials are followed, the information gained from properly conducted explanatory and pragmatic trials may be more valuable than that derivable from even the best controlled explanatory trial. The best information an explanatory trial can provide is the certainty that efficacy has been demonstrated by valid methods. From such results it may be expected that the treatment under investigation will work under real-world conditions.

## Advantages, limitations and challenges of explanatory and pragmatic trials

The advantages, limitations and challenges of explanatory and pragmatic trials can be summarized as follows. The results of a properly conducted pragmatic trial will provide information on the distribution of risks in the investigated patient groups. This information is not available in usual randomized trials. The pragmatic trial will also provide information on the effect of the selected intervention as compared to others. The interesting aspect is that all patients are stratified according to their chance to respond favorably to the investigated treatments or according to their risk of treatment failure. This means that the effects of treatments other than those being explicitly investigated serve as controls. An additional important difference between explanatory and pragmatic trials is related to the selection of patients. Patients who are eligible according to the inclusion criteria but are excluded in order to focus the target group or patients who refuse one of the investigated treatments, e.g. due to strong preferences, are not included in explanatory trials and therefore not considered in the evaluation of the results. In the pragmatic trials, we propose to include all eligible patients (i.e. patient who meet the inclusion criteria) and to evaluate these patients according to their baseline risks but regardless of preferences or the selected treatments.

The proposed design of non-randomized trials we have presented here should be interpreted neither to imply that explanatory, or “efficacy”, trials are irrelevant nor that randomized trials have no proper role in the evaluation of real world effectiveness. Rather, since they may confirm uniquely attributable effects of high cost, resource intensive, interventions, efficacy trials may be considered as an essential pre-requisite evidence of benefit by both, policy makers and practitioners, prior to the commencement of pragmatic trials. In other words, pragmatic trials, precisely because they demonstrate association with benefit in the real world context of multifactorial determinants of outcome, rather than specifically attributable effect, are premised on either proven or assumed efficacy of specific interventions being evaluated. In other words, in the setting of actual clinical care, all interventions are, to one extent or another, components of a ‘care bundle’, and it is the bundle, not the unique intervention, that is being evaluated for effectiveness. The concept of a ‘care bundle’ leads to the question on how many PCTs will be necessary to cover all important treatment modification that can definitely not be covered by specific RCTs. The answer may be that any treatment modification that is expected to generate better outcomes than an established therapy in any dimension (survival, quality of life including patient safety, monetary costs) should be supported by results of a PCT. In contrast to RCTs, it will be much easier to describe the most frequently used variations of a treatment protocol in a PCT because the sequence of step Nos. 4–6 (Table 1) of the treatment selection and the treatment reporting are different in explanatory and pragmatic trials. There will always be some variation in the adherence to a particular treatment protocol and it is always a matter of discussion which deviations can or cannot be tolerated in a trial. It is recommended in PCTs to describe prospectively the tolerated deviations from the investigated treatment protocols as well as the observed deviations from the investigated treatment options. As a large variation of individual treatment modifications will be observed, it is recommended to specify at least the most frequently used treatments in the third study arm.

The three-arm trial design that we have described is uniquely suited to contexts subject to tangible mechanisms of contributory effect of patient preference, what Brewin and Bradley (1989) refer to as “participative” interventions in which motivational variables play an inescapable role. In such settings, randomization and blinding inherently obscure the comparative effectiveness that a formal trial seeks to establish. In the case of interventions in which the participative component is concealed or absent, randomized trials that seek to address practical concerns may provide useful information that can guide policy and practice decisions.

## Conclusion

A significant step forward in the development of clinical trials can be taken, if we accept the principle that ‘form follows function’. This means that the problem to be solved in a research question is defined, the results are described in terms of either efficacy or effectiveness (but not as a mixture of both), and the limited consequences that can be derived from the obtained results are clear. When accepting the principle ‘form follows function’ for clinical trials it will be possible to shorten the development of new treatments from the bench to usual care and to generate reliable real world results when expectations, preferences and placebo effects will be included in the analysis.

A randomized clinical trial is a valuable tool but it can neither be used to solve any research question nor should it be regarded any longer as adequate in itself to address two different and essential types of questions: “Does a new principle work at all under ideal study conditions, i.e. is it efficacious” and “will the efficacious principle do under real world conditions what it promised to do under ideal study conditions, i.e. is it effective”?
